# Supplementary material for: Novel Drug-Like Somatostatin Receptor 4 Agonists are Potential Analgesics for Neuropathic Pain
Source: Int J Mol Sci. 2019 Dec 11;20(24):6245. doi: 10.3390/ijms20246245 (PMC6940912; doi:10.3390/ijms20246245)
Supplement: Supplementary file 1 [file ijms-20-06245-s001.zip › IJMS_Supplementary_Materials.docx]

**Supplementary Materials**

Novel drug-like somatostatin 4 receptor agonists are potential analgesics for neuropathic pain

**Boglárka Kántás^1,2^, Rita Börzsei^3^, Éva Szőke^4,5^, Péter Bánhegyi^6^, Ádám Horváth^7,8^, Ágnes Hunyady^9,10^, Éva Borbély^11,12^, Csaba Hetényi^13^, Erika Pintér^14,15^, Zsuzsanna Helyes^16,17^***

Detailed preparation methods of the compounds

1. 4-Chloro-7-benzyl-5,6-dimethyl-pyrrolo[2,3-d]pyrimidine intermediate


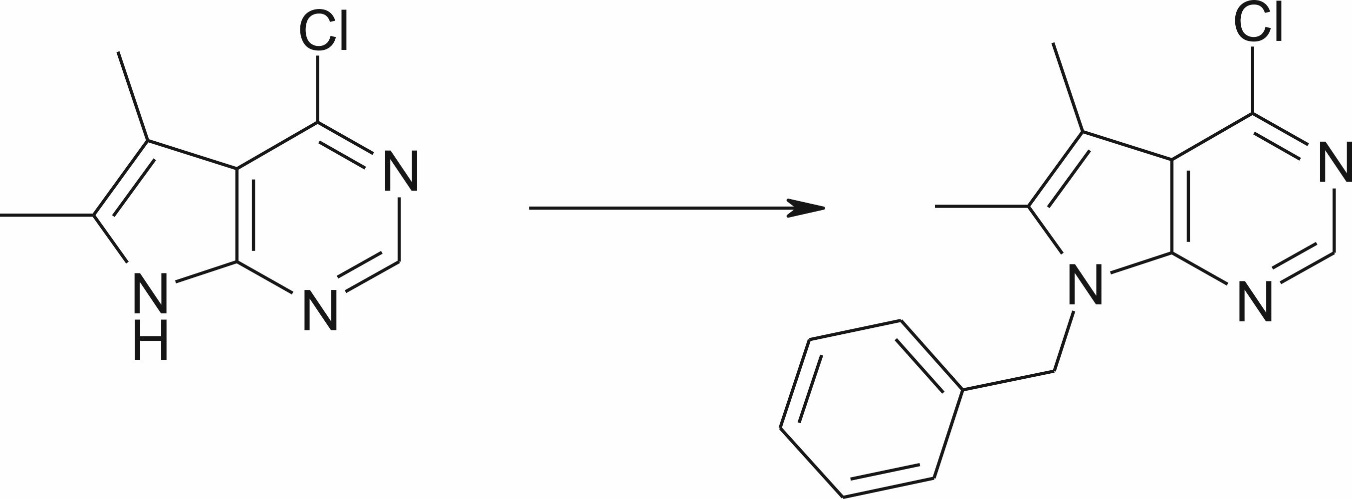


**Scheme S I.**

910 mg (5 mmol) 4-chloro-5,6-dimethyl-7H-pyrrolo[2,3-d]pyrimidine was solved in 6 ml abs. N,N-dimethylformamide. The solution was cooled down to 0°C, and 240 mg (6 mmol) sodium hydride (60 % dispersion in mineral oil) was added in small amounts. After the addition the reaction mixture was stirred for 30 minutes at room temperature, then 654 µl (5.5 mmol) benzyl bromide was added, and the mixture was stirred overnight. After the starting chlorine compound disappeared by TLC (eluent: chloroform/methanol 10/1) the mixture was diluted with 60 ml water. The product was extracted with 3x25 ml ethyl acetate. The organic layers were separated, combined, and dried over Na_2_SO_4_. The solvent was removed under vacuum, the remaining oil was treated with diisopropyl-ether to obtain the solid product which was used for the next step without further purification.

Yield: 1.13 g (83%) yellow material.

1. 4-Methoxy-benzyl-[7-benzyl-5,6-dimethyl-7H-pyrrolo[2,3-d]pyrimidin-4-yl]-amine (Compound 4)


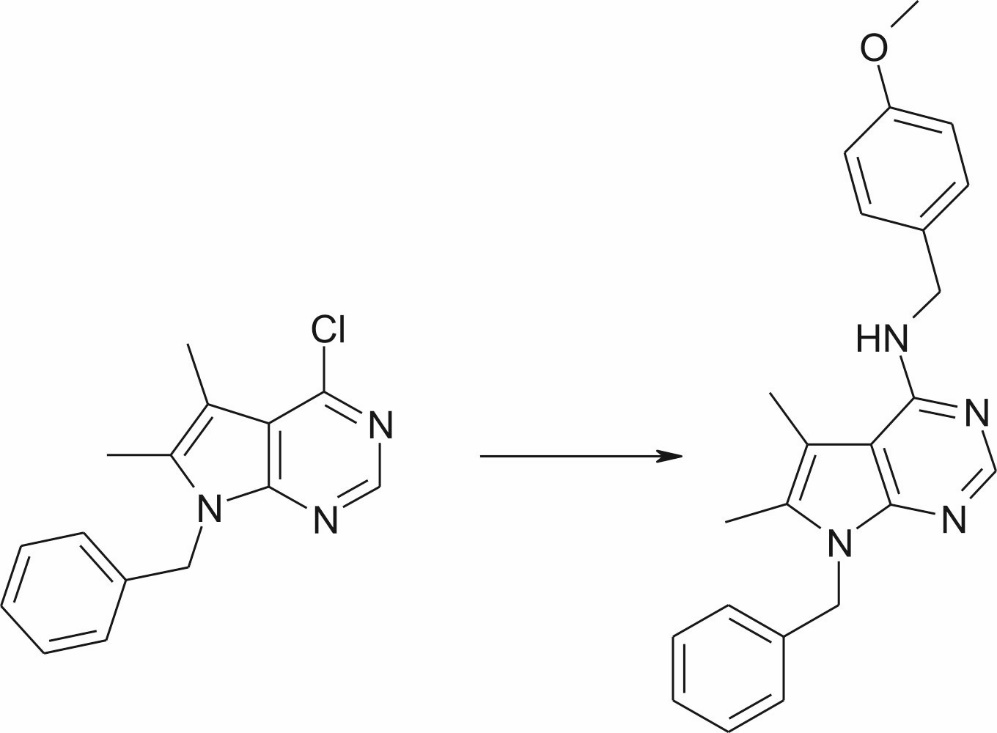


**Scheme S II.**

50 mg (0.18 mmol) 4-chloro-7-benzyl-5,6-dimethyl-pyrrolo[2,3-d]pyrimidine and 60 µl (0.46 mmol) 4-methoxy-benzylamine were dissolved in 1 ml dimethyl sulfoxide. The solution was stirred at 100°C until the starting material disappeared by TLC (eluent: chloroform/methanol 10/1). The reaction mixture was allowed to cool down to room temperature and diluted with 1N HCl solution. The solid was filtered off and purified by chromatography to obtain the pure product.

Yield: 15 mg (22%) white solid.

LCMS: 3.42 min

1H-NMR: 8.20(s,1H), 7.85-8.10(bs,1H), 7.31(m,4H), 7.02(d.2H), 6.90(t,1H), 6.88(d.1H), 6.81(m,1H), 5.41(s,2H), 3.81(s,2H), 3.71(s,3H), 2.31(s.3H), 2.17(s,3H)

1. (7-Benzyl-5,6-dimethyl-7H-pyrrolo[2,3-d]pyrimidin-4-yl)-[2-(4-methoxy-phenyl)-ethyl]-amine (Compound 2)


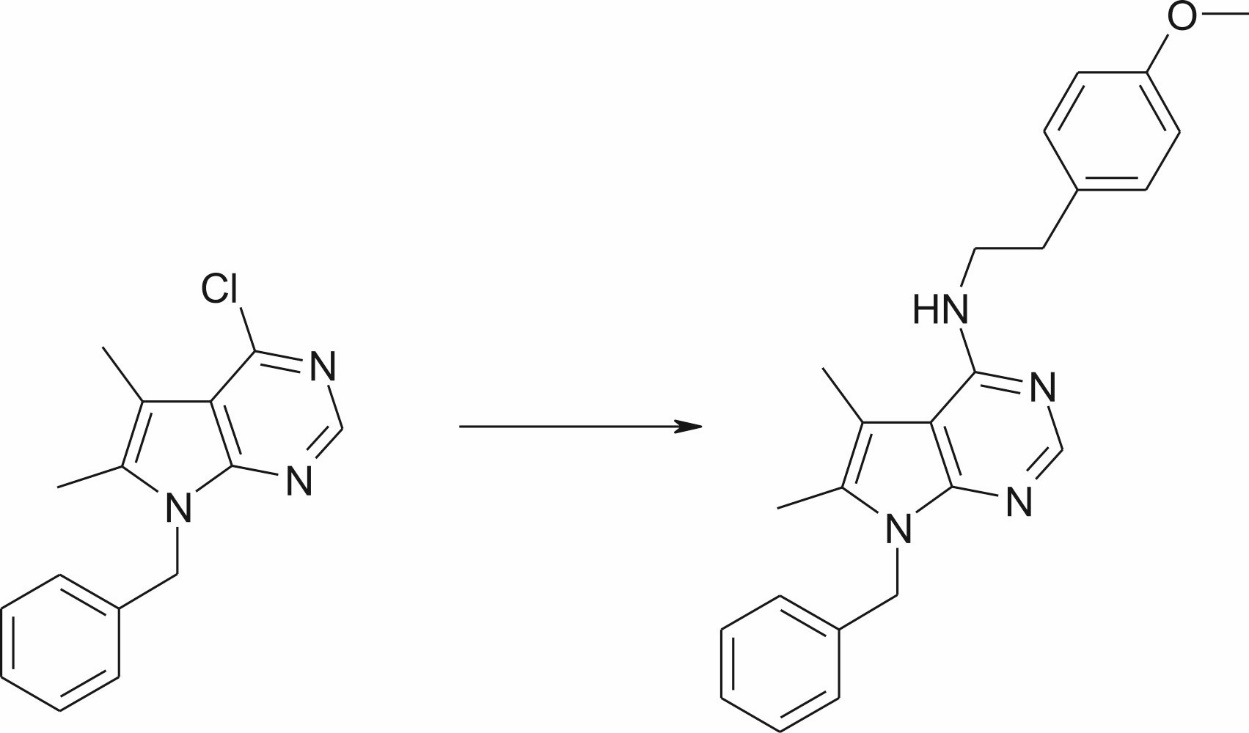


**Scheme S III.**

50 mg (0.18 mmol) 4-chloro-7-benzyl-5,6-dimethyl-pyrrolo[2,3-d]pyrimidine and 70 µl (0.46 mmol) 4-methoxy-phelyl-ethylamine were solved in 1 ml dimethyl sulfoxide. The solution was stirred at 100 °C until the starting material disappeared by TLC (eluent: chloroform/methanol 10/1). The reaction mixture was allowed to cool to room temperature and was diluted with saturated NH_4_Cl solution. The solid was filtered off and purified by chromatography to obtain the pure product.

Yield: 19 mg (27%) white solid.

LCMS: 3.44 min

1H-NMR: 8.27(s,1H), 8.00(bs,1H), 7.26(m,4H), 7.07(d.2H), 6.96(t,1H), 6.90(d.1H), 6.79(m,1H), 5.45(s,2H), 3.86(q,2H), 3.73(s,3H), 2.98(t,2H), 2.33(s.3H), 2.20(s,3H)

1. 7-Benzyl-4-chloro-7H-pyrrolo[2,3-D] pyrimidine


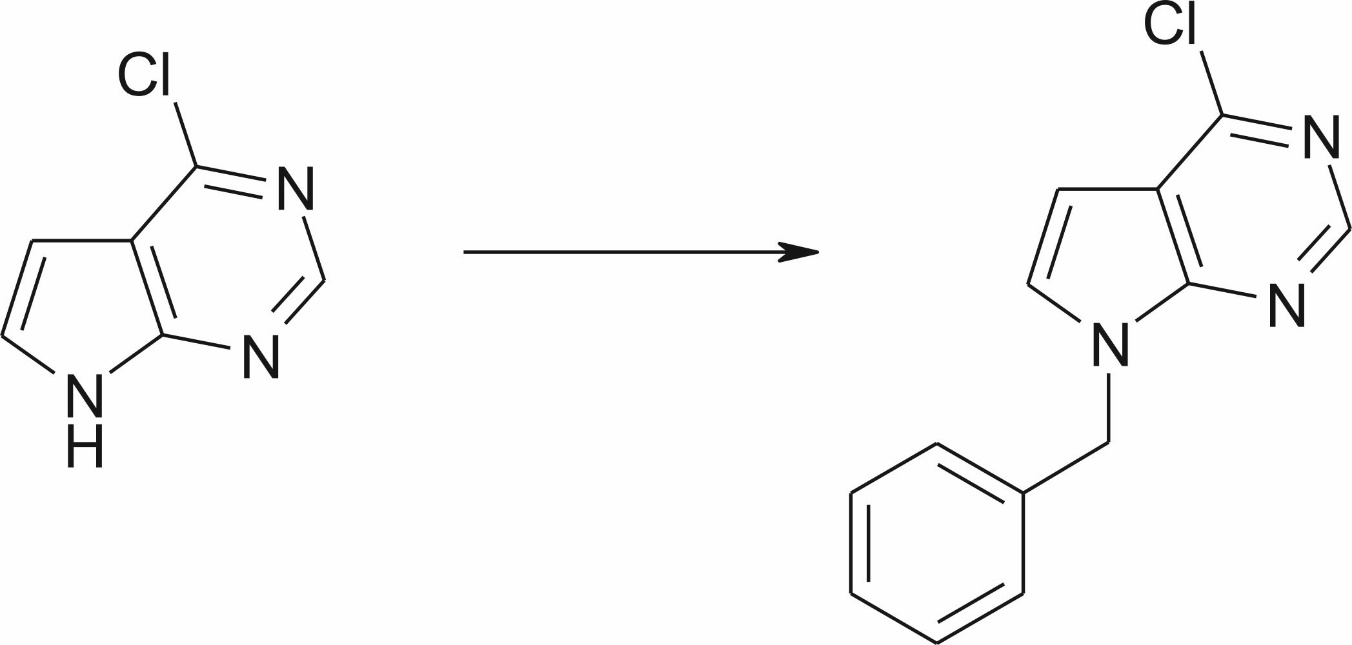


**Scheme S IV.**

670 mg (5 mmol) 4-chloro-7H-pyrrolo[2,3-d]pyrimidine was solved in 5 ml abs. N,N-dimethylformamide. The solution was cooled down to 0°C, and 240 mg (6 mmol) sodium hydride (60% dispersion in mineral oil) was added in small amounts. After the addition the reaction mixture was stirred for 30 minutes at room temperature, then 654 µl (5.5 mmol) benzyl bromide was added, and the reaction mixture was stirred overnight. After the starting chlorine compound disappeared by TLC (eluent: chloroform/methanol 10/1) the mixture was diluted with 60 ml water. The product was extracted with 3x25 ml ethyl acetate. The organic layers were separated, combined, and dried over Na_2_SO_4_. The solvent was removed under vacuum, the remaining oil was treated with diisopropyl-ether to obtain the solid product which was used for the next step without further purification.

Yield: 926 mg (76%) yellow material.

1. Benzyl-[7-benzyl-5,6-dimethyl-7H-pyrrolo[2,3-d]pyrimidin-4-yl]-amine (Compound 3)


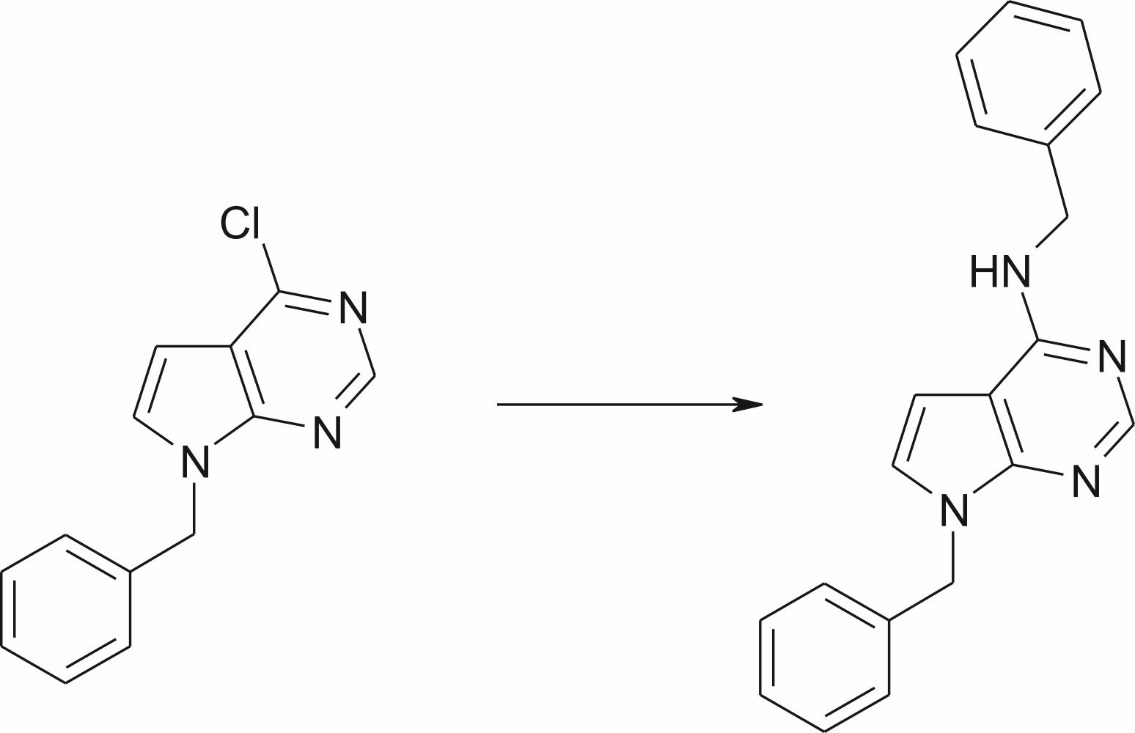


**Scheme S V.**

122 mg (0.5 mmol) 7-benzyl-4-chloro-7H-pyrrolo[2,3-D]pyrimidine and 164 µl (1.5 mmol) benzylamine were stirred in 1.5 ml dimethyl sulfoxide at 100°C for 12 hours. The reaction mixture was poured onto 15 ml 0.5N HCl, the solid product was filtered off, washed with water and n-hexane, then dried.

Yield: 85 mg (49%) off-white material.

LCMS: 3.27 min

NMR: 9.81(bs,1H), 8.29(s,1H), 7.54(d,1H), 7.31-7.20(m,10H), 7.10(d,1H), 5.33(s,2H), 3.04(s,2H)

1. (7-Benzyl-7H-pyrrolo[2,3-d]pyrimidin-4-yl)-phenethyl-amine (Compound 1)


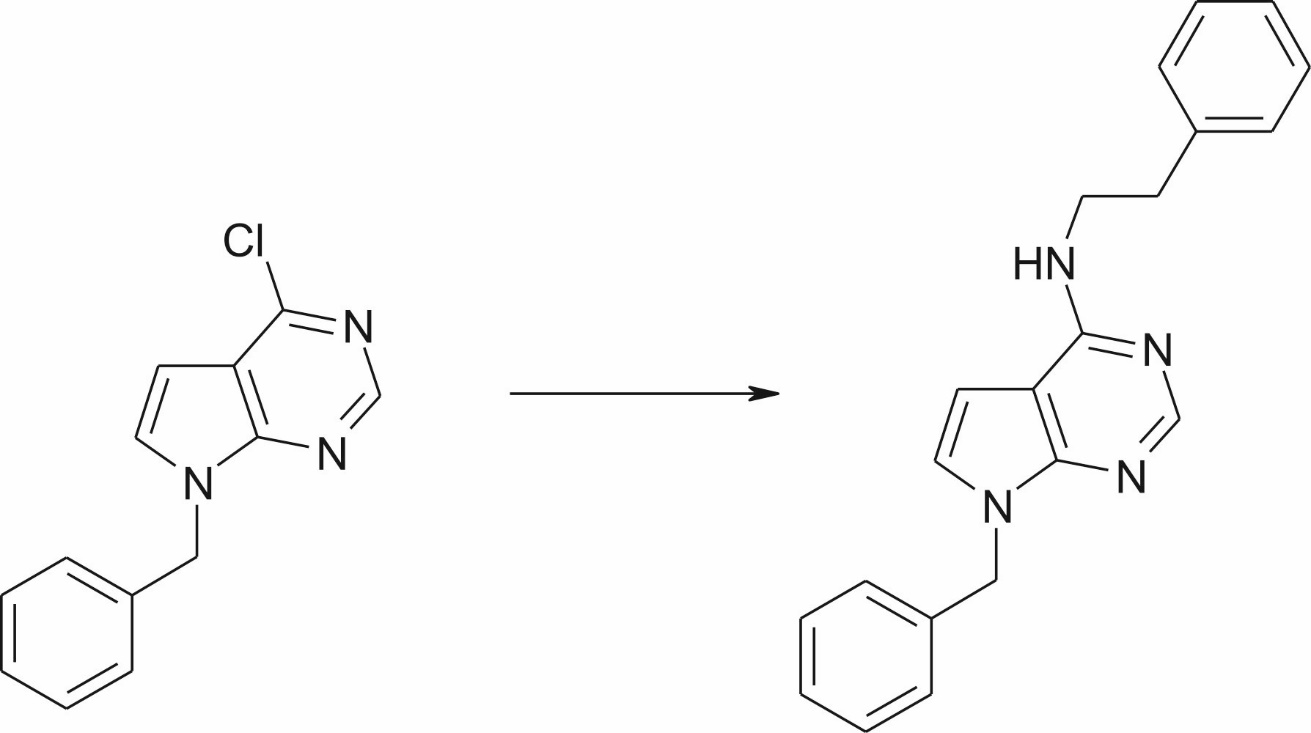


**Scheme S VI.**

44 mg (0.18 mmol) 4-chloro-7-benzyl-pyrrolo[2,3-d]pyrimidine and 58 µl (0.46 mmol) 4-methoxy-phelyl-ethylamine were solved in 1 ml dimethyl sulfoxide. The solution was stirred at 100°C until the starting material disappeared by TLC (eluent: chloroform/methanol 10/1). The reaction mixture was allowed to cool to room temperature and was diluted with saturated NH_4_Cl solution. The solid was filtered off and purified by chromatography to obtain the pure product.

Yield: 19 mg (32%) white material.

LCMS: 3.20 min

1H-NMR: 9.78(bs,1H), 8.33(s,1H), 7.56(d,1H), 7.37-7.22(m,10H), 7.04(d,1H), 5.45(s,2H), 3.81(q,2H), 3.00(t,2H)
