# Supplementary figures and images for: Novel Drug-Like Somatostatin Receptor 4 Agonists are Potential Analgesics for Neuropathic Pain
Source: Int J Mol Sci. 2019 Dec 11;20(24):6245. doi: 10.3390/ijms20246245 (PMC6940912; doi:10.3390/ijms20246245)

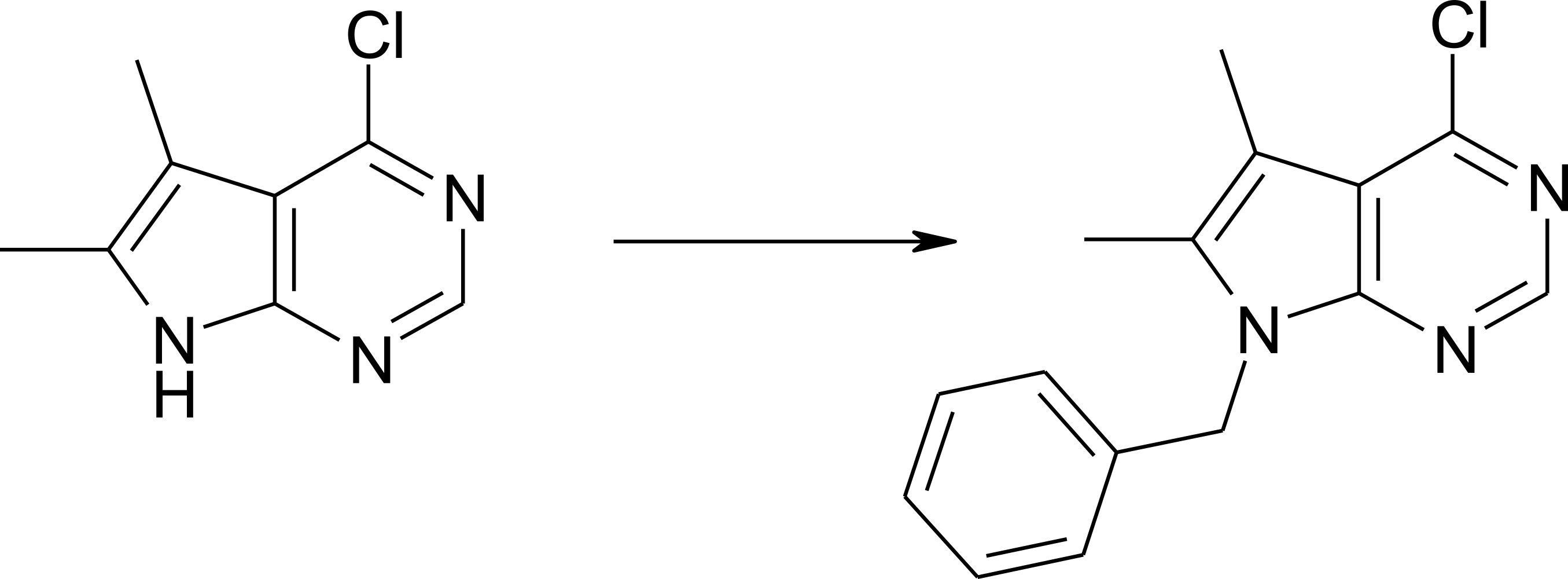

Supplement: Supplementary file 1 [file ijms-20-06245-s001.zip › IJMS_Supp_Mat/Scheme_SI.jpg]

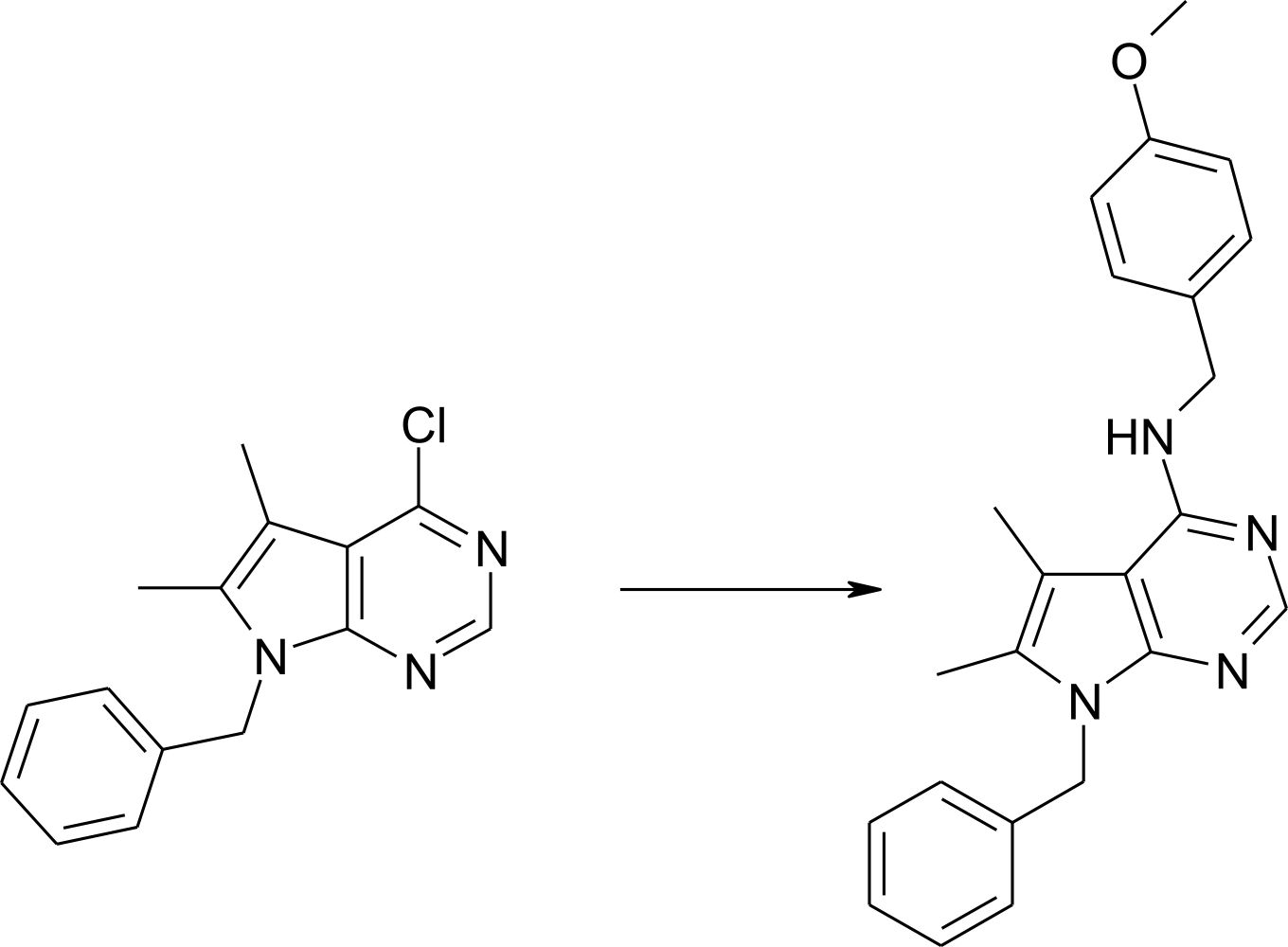

Supplement: Supplementary file 1 [file ijms-20-06245-s001.zip › IJMS_Supp_Mat/Scheme_SII.jpg]

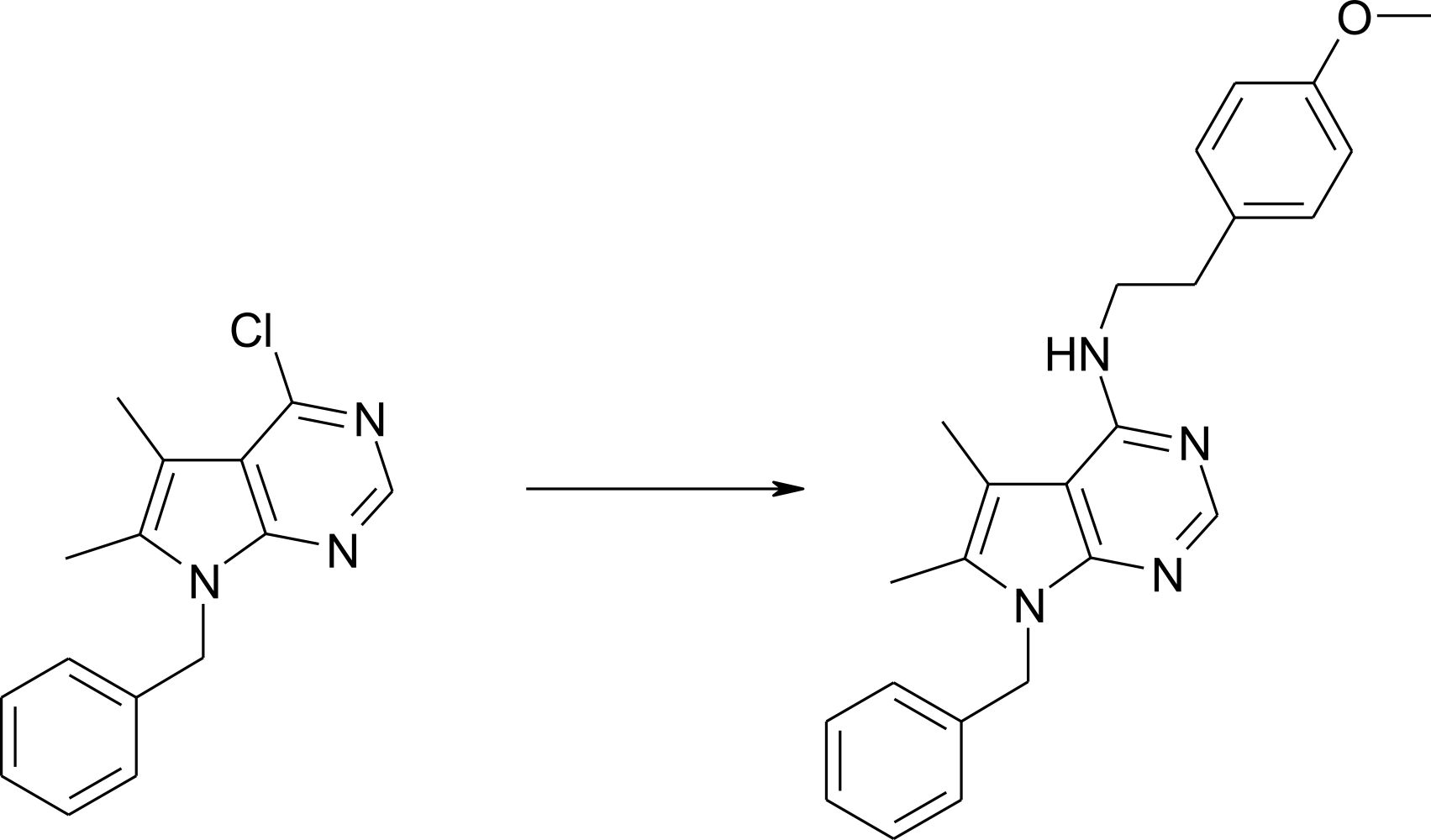

Supplement: Supplementary file 1 [file ijms-20-06245-s001.zip › IJMS_Supp_Mat/Scheme_SIII.jpg]

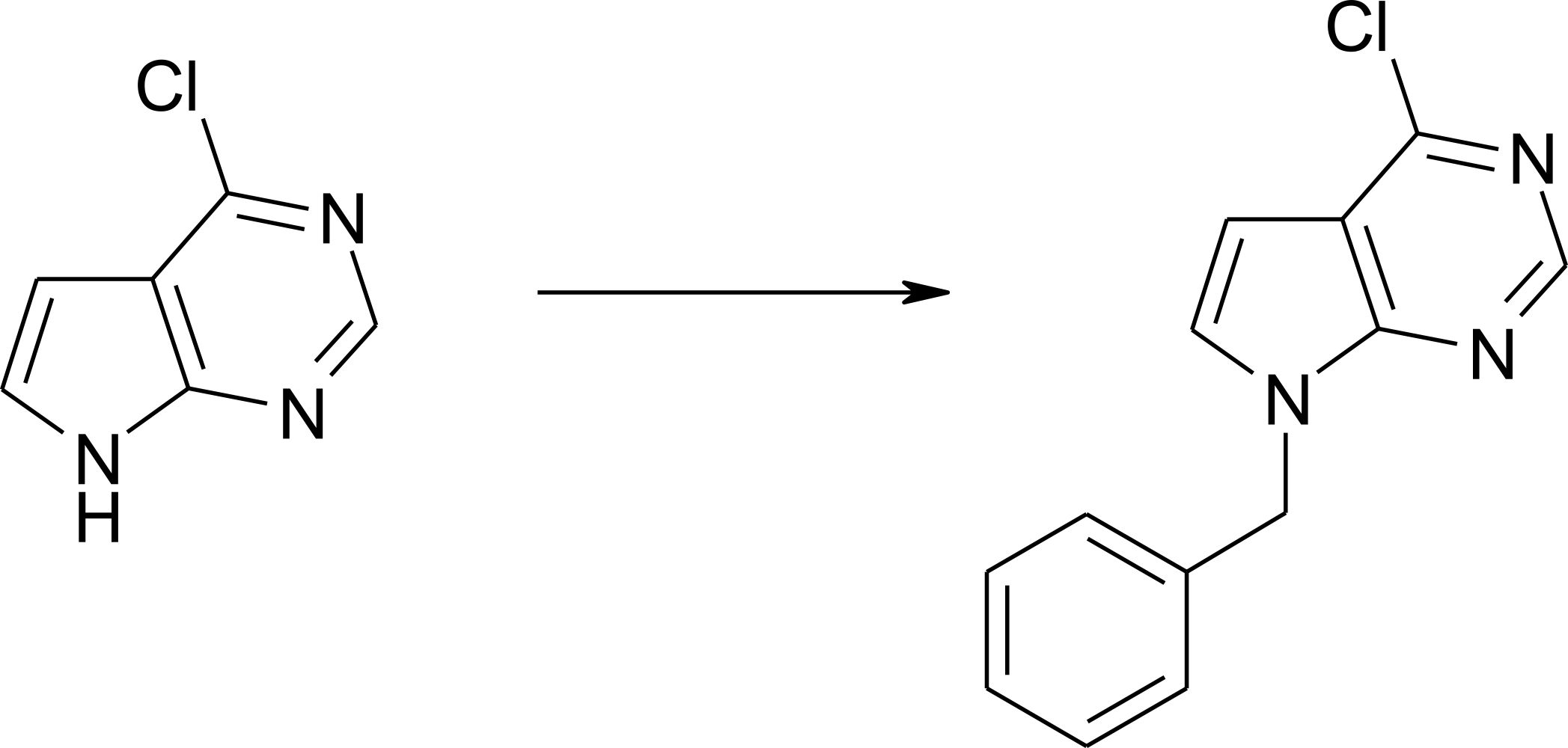

Supplement: Supplementary file 1 [file ijms-20-06245-s001.zip › IJMS_Supp_Mat/Scheme_SIV.jpg]

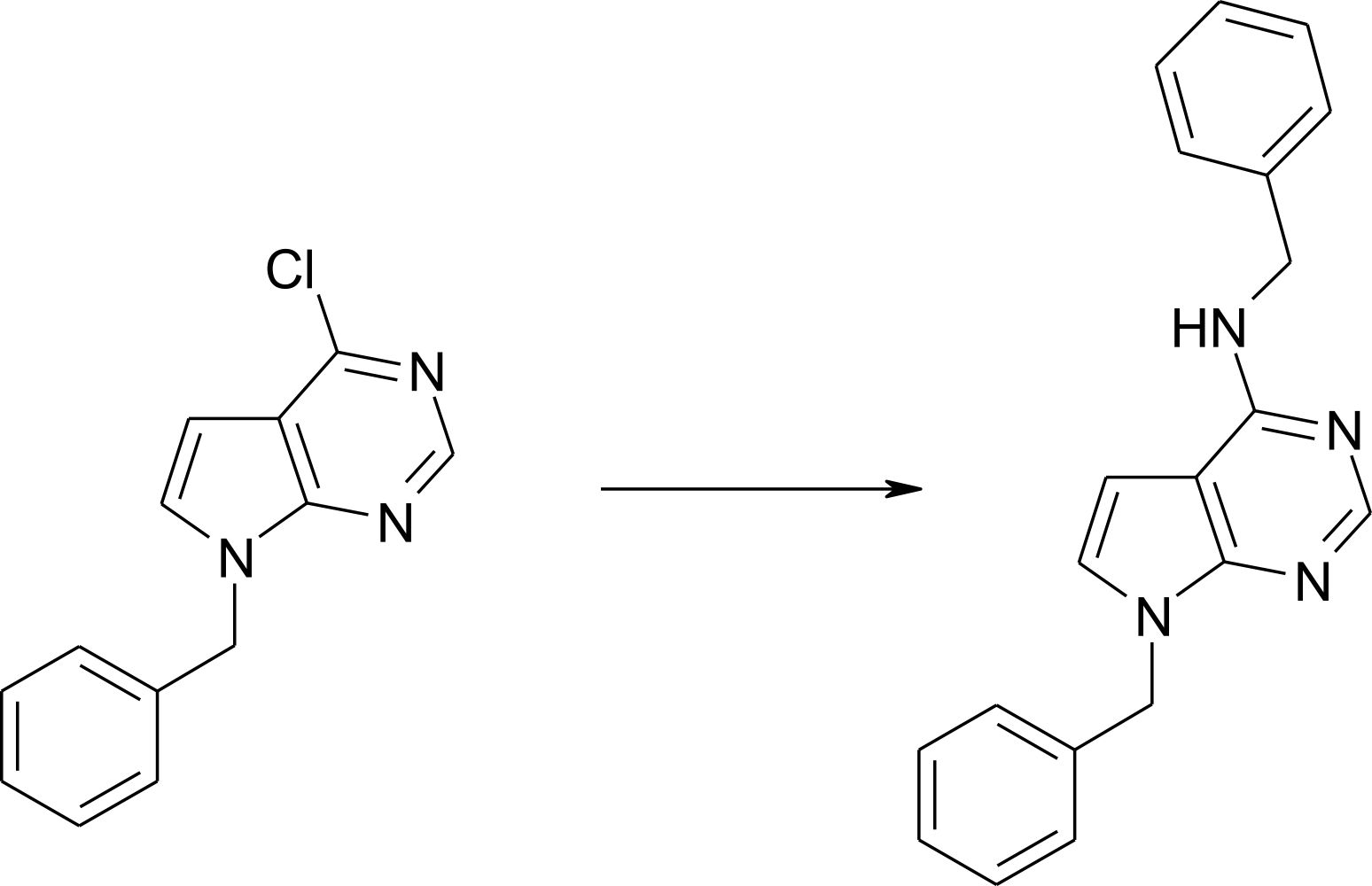

Supplement: Supplementary file 1 [file ijms-20-06245-s001.zip › IJMS_Supp_Mat/Scheme_SV.jpg]

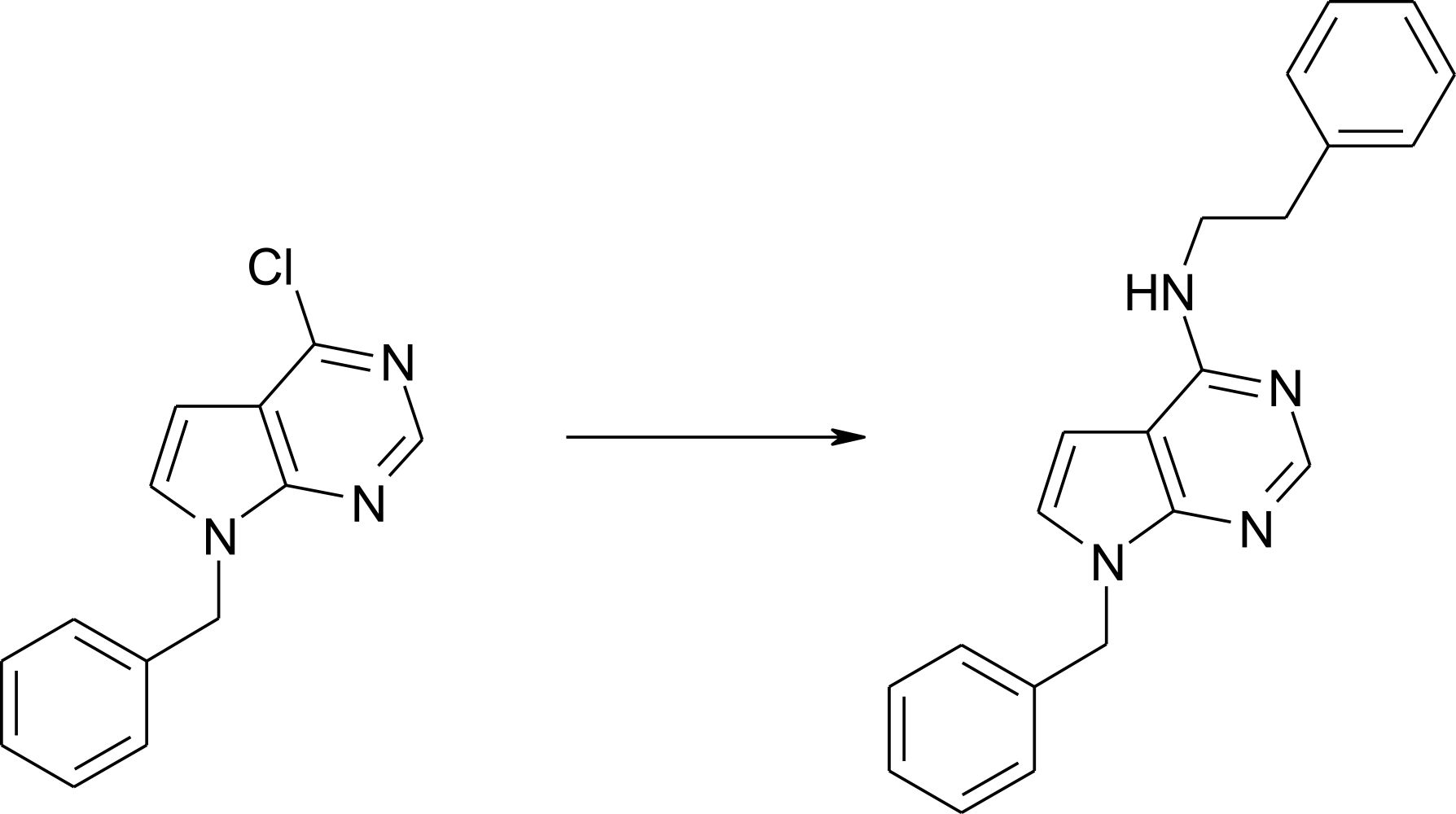

Supplement: Supplementary file 1 [file ijms-20-06245-s001.zip › IJMS_Supp_Mat/Scheme_SVI.jpg]
